# A Comprehensive Review on Novel Lipid-Based Nano Drug Delivery

**DOI:** 10.34172/apb.2024.012

**Published:** 2023-10-14

**Authors:** Sonam Suresh Godase, Nilesh Shrikant Kulkarni, Shashikant Nivrutti Dhole

**Affiliations:** Department of Pharmaceutics, PES Modern college of Pharmacy (for ladies) Moshi, Pune. Affiliated to Savitribai Phule Pune University, Pune, Maharashtra, India.

**Keywords:** Novel Drug Delivery System, BCS classification, Liposome, Niosomes, Solid lipid nanoparticles, Nanochochleats

## Abstract

Novel drug delivery system opens the doors towards nano/micro formulation strategies to overcome the challenges associated with the poorly soluble and permeable drugs. Lipid based nanoparticles are widely accepted that includes liposomes, niosomes and micelles which are FDA approved. Such lipid based drug delivery allows delivery for natural phytoconstituents, biopharmaceutical classification system (BCS) class II and class IV drugs are effectively delivered to improve its solubility, permeability and bioavailability. The article provides the recent advances and application of lipid based dosage form for improvement of therapeutic efficacy.

## Introduction

 Novel drug delivery system opens the doors towards Nano/Micro formulation strategies to overcome the challenges associated with the biopharmaceutical classification system (BCS) class II and class IV drugs.^1^ Such medication or drug delivery targets the drug at required site that too in low concentration and improves therapeutic efficiency. Novel drug delivery system includes microparticles, nanoparticles such as lipid based liposomes, niosomes, phytosomes, micelles, hydrogels, quantum dots, nanotubes, dendrimers etc.^2^ Nanoparticulate drug delivery system have particle size which ranges between 1 to 100 nm. The drug movement across the barrier will get improved due to development of nanosized particulate system.^3^ Nanomaterials have wide application in the treatment and diagnostic purpose.^4,5^

 Currently lipid based dosage forms are popular that includes liposomes, niosomes, micelles etc which are FDA approved. Such lipid based drug delivery systems have found to be effective for natural phytoconstituents and inorganic particles like gold.^6^ The advantages of lipid based novel drug delivery system are associated with the majority of drugs.

 Reasons for application of novel drug delivery system for BCS class II and IV drugs.^7-11^

1. Poor solubility and poor permeability of drug.
2. Decrease in size of particle leads to increase in effective surface area which ultimately improves dissolution rate of poorly soluble drugs.
3. Nanomaterials are being used in many different biological and medical fields because they reframe optical, electrical, chemical and physical properties.
4. Increases mobility of particle that helps to increase bioavailability.
5. Nanomaterials have application in targeted and controlled delivery of biopharmaceuticals.
6. Due to nanosized structure, it can easily cross mucosal membrane whereas Microsystems has capacity to cross epithelial lining.
7. Increased drug therapeutics efficacy and reduced side effects.
8. Protection of drug from first pass metabolism and enzymatic degradation.

## Solubility and permeability

 Solubility is one of the key parameter that directly affects the activity and bioavailability of drug. The variety of factors that has influence on solubility of the drugs are pKa of drug, pH at gastrointestinal tract (GIT), presence of luminal pH.^12,13^ Physiological and physicochemical factors have influence on drug solubility.^14,15^

 Solubility depends on chemical, electrical, structural properties of the solute and interaction between solute solvent. The USP 38, European pharmacopoeia categorized solubility in seven different group.^16^ Biopharmaceutics classification system was developed by Amidon et al in 1995. The BCS classification has application for the development of immediate release oral dosage forms. The drugs will be classified into four classes.^17-19^ Solubility and permeability improvement for BCS class II and BCS class IV drugs respectively has a major obstacle for the formulation scientist (Table 1). There are various approaches are reported till today to enhance the solubility for such drugs. Permeability study also shows the movement of drug into the circulatory system through GIT.

**Table 1:** BCS Classification

**Class**	**Solubility**	**Permeability**	**Example**
Class I	High	High	Metoprolol, diltiazem, verapamil, propranolol etc.
Class II	Low	High	Ibuprofen, ketoprofen, carvedilol, ketoconazole, fenofibrate etc.
Class III	High	Low	Cimetidine, ranitidine, acyclovir, neomycin B, atenolol, captopril.
Class IV	Low	Low	Hydrochlorothiazide, taxol, furosemide.

 The BCS class II drugs will be classified into subclasses considering the acidic and basic strength (Table 2).^20-22^ Variation in pH environment in GIT has influence on drug solubility for BCS class II drugs.

**Table 2:** BCS Sub classification

**Class II**	**Solubility**		**Example**
	**Gastric pH solubility**	**Instestinal pH solubility**	
Class II a (Weakly acidic drugs)	Low	Dissolve quickly	Ibuprofen, ketoprofen, flurbiprofen, naproxen, rifampicin etc
Class II b (Weakly basic drugs)	High	Precipitate	Carvedilol, ketoconazole, ibuprofen, ketoprofen etc
Class II c (Neutral drugs)	No dependent on pH change		Fenofibrate etc.

 BCS classification allows the formulator to correlate the physicochemical properties of drug and its solubility, permeability to make a judgment on bioavailability. It reduces the time, cost of drug delivery and development. It is approved by US Food and Drug Administration (USFDA). The regulatory agencies such as European Medicine Agency (EMEA) and World Health Organization (WHO) for bioavailability/bioequivalence standards for approval of drug product and gives directions for In-Vitro, In-vivo dissolution study.^23,24^

 Basic fundamentals of BCS classification are the three dimensionless numbers as dose number, absorption number and dissolution number which calculates the amount of drug.^25^

 Dose number (high solubility): when the highest clinical dose is dissolved in 250 mL buffer at all pH values within the range 1–7.5.

 Permeability: High permeability means the drug product is stable in GIT and drug absorption is greater than 90% of the given dose. Permeability is defined as passage or movement from site of administration (Gastrointestinal track) to the systemic circulation across the biological membranes is called permeability. Permeability depends upon the absorption of drug and absorption is depend upon various properties of drug, receptors, biological membranes, types of transport etc.

## Types of lipid based nano drug delivery system

**Figure 1:**
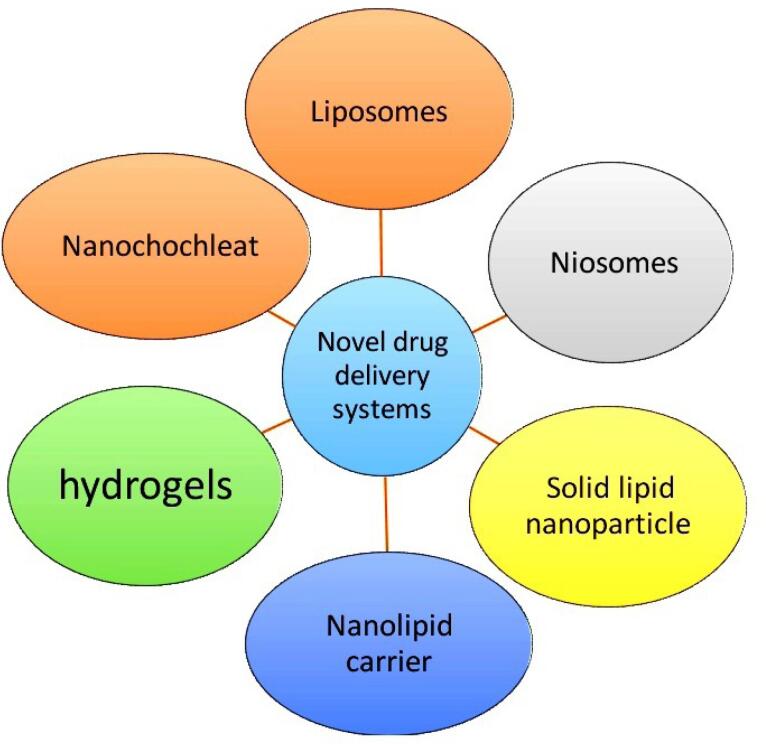
Types of novel drug delivery system

 The major obstacle for the drugs to develop into dosage form is associated with poor aqueous solubility, poor permeability, poor absorption, extensive first pass metabolism, systemic metabolism and efflux proteins (P-glycoprotein).^26^ It is important for further clinical improvements of drugs. Researchers were tried with variety of techniques to overcome these issues which includes Lipid based drug delivery systems, Polymer based drug delivery system, Nanocarriers, Nanocrystals, liquisolid technology, solid dispersions etc (Figure 1). Amongst these techniques lipid based nanoparticulate formulation was found to be beneficial.

## Liposomes

 Liposomes are the spherical vesicles made up of amphiphilic phospholipids. Phospholipids has capability to encloses both hydrophilic and hydrophobic drugs and possess property to self assemble.^27^

## Mechanism of liposome formation

**Figure 2:**
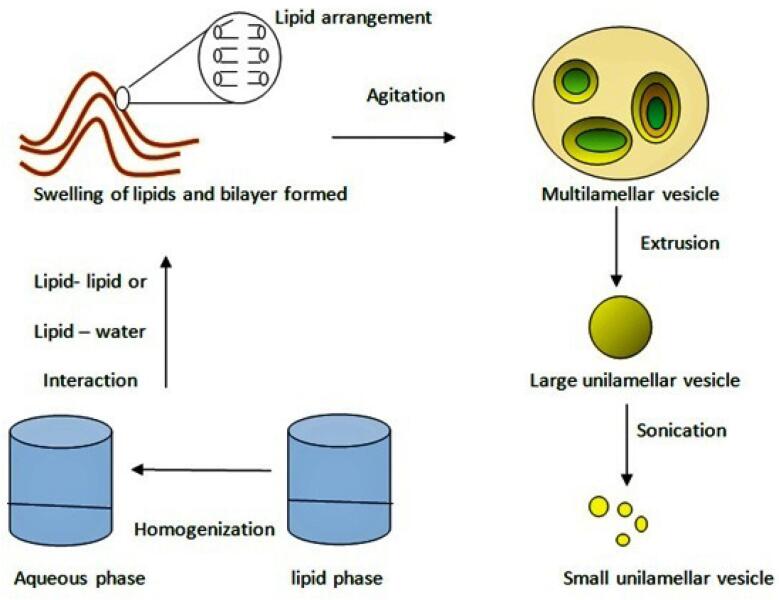
Mechanism of liposome formation

 The lipids phase is added into the aqueous phase. It forms bilayers by hydrophobic interaction or hydrophilic interaction between lipid–lipid or lipid–water molecules (Figure 2). These formed lipid layers are set as vesicles by external energy such as sonication, homogenization, heating, freezing etc.

## Classification of liposomes

**Figure 3:**
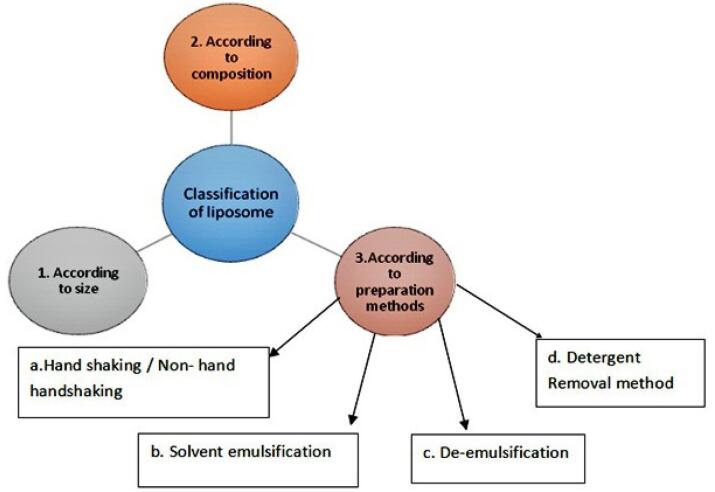
Classification of liposomes

 The liposomes will be classified based on material used for the preparation, types of lipid or combination of lipids used, based on method of preparation techniques and depending upon the size of vesicles formed (Figure 3).

A. According to size and shape of liposome: Liposomes were classified according to the size, number of bilayers formed in particle and according to their pattern. They are classified as multilamellar large vesicle which is greater than 0.5 µm size. Multilamellar liposomes are those which are with a number of lipidic bilayers. Oligolamellar liposomes means vesicles are same as that of multilamellar. Oligolamellar vesicles are made up with 2 to 5 lipid bilayers. More than 5 lipid bilayer considering as multilamellar vesicles. Unilamellar vesicles (ULV), small unilamellar vesicles (SUV) and large unilamellar vesicles (LUV) possess similarity in structure but varies in size (Table 3). 

**Table 3:** Types of liposomes according to size

**Type**	**Size**
Multilamellar large vesicle	> 0.5 µm
Oligolamellar vesicles (OLV)	0.1-1.0 µm
Unilamellar vesicles (ULV)	All size range (o.1 nm to 1000 µm)
small unilamellar vesicle (SUV)	20-100 nm
Large unilamellar vesicles (LUV)	> 100 nm
Giant unilamellar vesicles (GUV)	> 1.0 µm
Multivariant vesicle	> 1.0 µm

B. Based on composition: According to the source of lipids used in preparation of liposome (Table 4). 

**Table 4:** Types of liposomes based on lipid composition

**Type of composition**	**Application**	**Examples of lipids**
Conventional liposomes	To improve drug delivery	Neutral or negatively charged lipids examples phospholipid lecithin, glycerol, fatty acids etc^28^
pH sensitive liposome	According to pH intracellular drug delivery	Neutral to slightly alkaline pH to acidic lipids examples phosphatidyl ethanolamine, dioleoyl phasphatidyl ethanolamine etc^29^
Cationic liposomes (Positively charged head groups) or lipoplexes	For delivery of negatively charged macromolecules (DNA, RNA)	DOTAP (1,2–dioleoyl-3,3-trimethyl ammonium-propane (chloride salt), DOTMA(1,2,3–dioleoloxy) 3,3 trimethyl ammonpropane etc^30^
Stealth liposomes or long circulating or PEGylated liposomes	To avoid immune system and extracellular delivery of drug	Includes synthetic polymers in liposome composition example Polyethylene glycol^31^
Immunoliposome	Cell specific binding with avoiding immune system	Antibodies attached to conventional liposomes^30^
Magnetic liposomes	Use by external vibrating magnetic field at deliberate site for immediate release on site	Phosphatidylcholine, Cholesterol, linear chain aldehyde and colloidal particles of magnetic iron oxide^31^
Temperature sensitive or heat sensitive	Liposomes release drugs at target cell according to temperature or heat change	Dipalmitoyl phosphatidylcholine^28^

C. Based on method for preparation of liposome: Various methods are reported for the preparation of liposome as mechanical dispersion, solvent dispersion, de-emulsification, detergent removal method (Table 5). 

**Table 5:** Types of methods of preparation of liposomes

**Name of method**		**Instruments used**
Mechanical dispersion: Process: Co-dissolving lipids in organic solvent, Organic solvent is removed by film deposition under vacuum.	Hand shaking/Non hand shaking	
	Sonication (bath sonicator or probe sonicator)	Ultra sonicator^32-34^
	Micro emulsification	Microfluidizer pump^35,36^
	Extrusion technique	Extruder^37,38^
Solvent dispersion: Process: Lipids are dissolved in organic solvent then add into aqueous phase containing drug.	Ethanol injection (water miscible solvent)	Fine needle^39^
	Ether injection (water immiscible solvent)	Fine needle^40^
	Rapid solvent exchange	Narrow needle^41^
De-emulsification: Process: breakdown of large emulsion vesicles that have capacity to reform when broken down.	Reverse phase evaporation technique	Evaporator^42^
Detergent removal method: Process: micelles are formed with the help of detergents.	Dialysis	Membranes^43^
	Column chromatography	Columns^43^

## Niosomes

 Niosomes are the non-ionic surfactants containing liposomes. Surfactants such as fatty alcohol, esters and copolymers are used in the development of niosome formulation. Niosomes formulation contains surfactant.^44,45^

 The main component is surfactant. The surfactants possess both hydrophilic and hydrophobic groups and hence these are widely accepted (Table 6). According to head group properties, surfactants are classified as anionic, cationic, amphoteric and nonionic. Nonionic surfactant is mostly used because they are more stable, less toxic and compatible.^46^

**Table 6:** Examples of niosome prepared by film hydration Technique

**Technique of preparation**	**Excipients used**	**Compound/ Drug used**
Thin film hydration (sonication)	Tween 80, Tween 20, Phosphate buffer pH 7, Cholesterol	Curcumin^47^
Thin film hydration	Chloroform, Methanol, Span80, Dicetyl phosphate	Curcumin^48^
Reverse phase evaporation	Span 60, DMSO, cholesterol	Growth factor^49^
Thin film hydration (evaporator)	GMS, Cholesterol, Glucose, Sodium chloride, Tween 80, MYRJ 49	Ginkgolide^50^

###  Advantages

1. Designed for drugs which has poor absorption to enhance bioavailability.
2. Solubility/ Permeability is enhanced as niosomeal drug delivery crosses anatomical barriers of GIT via transcytosis of M cells of Peyer’s patches in intestine.
3. Niosomes has capacity to release drugs in the gradual and controlled manner.
4. Niosomes are easily modified due to presence of hydrophilic and lipophilic head groups.

###  Disadvantages

1. Physical instability (aggregation, fusion)
2. Hydrolysis of entrapped drug.
3. Leaking and leaching of an entrapped.

## Solid lipid nanoparticles

 The solid lipid nanoparticles (SLNs) are need to be developed to overcome drawbacks associated with traditional colloidal systems such as emulsions, liposomes, polymeric nanoparticles The SLNs are composed of physiological lipids like glycerides of fatty acids which possess biocompatibility and biodegradability. SLNs overcomes the drawbacks associated with traditional colloidal systems as complicated preparation methods, low entrapment efficiency, difficult large scales manufacturing.^51^

 Key ingredients to be used for formulation of SLNs includes:

1. Lipids – triglycerides, partial glycerides
2. fatty acids
3. Steroids
4. Waxes

**Figure 4:**
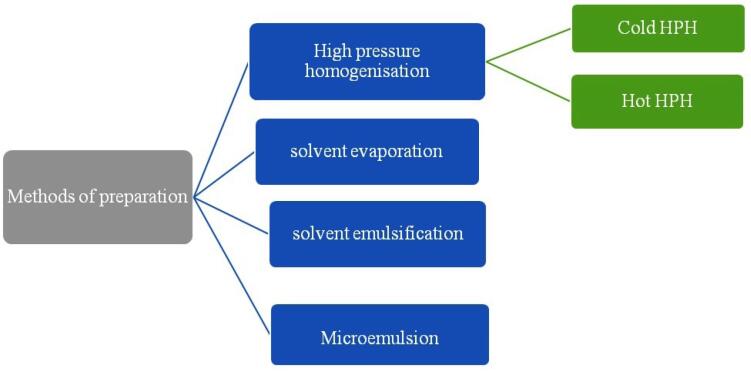
Methods of preparation of solid lipid nanoparticles

Different methods of preparation for solid-lipid Nanoparticle are reported (Figure 4).^52^

###  A. High pressure homogenization (HPH)

 HPH is most widely used and accepted technique used in pharmaceutical industries. In the high pressure homogenizer, liquid phase is need to passed with high pressure through narrow orifice of micron or submicron size. This leads to reduction in particle size. HPH process is of two types as hot homogenization and cold homogenization. For both the types drug is to be dissolved in the lipids and dispersion is made. Afterward according to method temperature is need to be maintained.

####  I. Hot homogenization method

**Figure 5:**
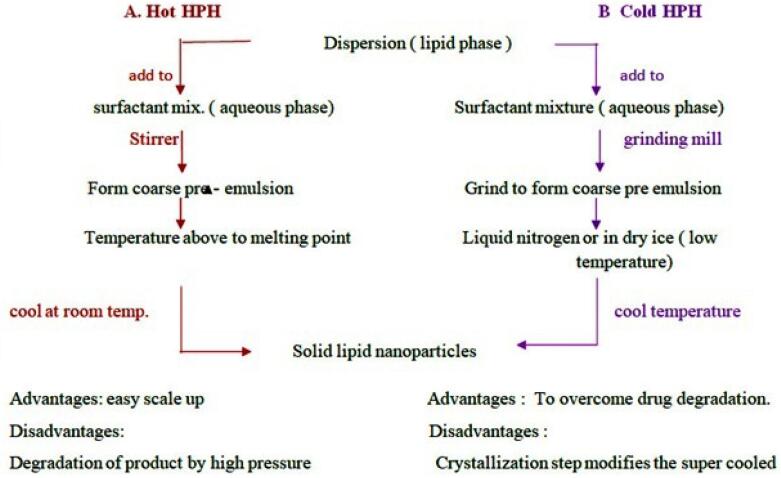
Types of high pressure homogenization method

 The hot homogenization method includes temperature which is more than the melting point of the lipid. Lipid is allowed to melt and into molten lipid drug is added. This process makes microemulsion also called as pre-emulsion which is maintained at high temperature and mixed with the aqueous phase with surfactants (Figure 5).^53^

####  II. Cold homogenization method

 The heat sensitive drugs undergo degradation at high temperature in hot homogenization method. To improve drug stability cold homogenization method is preferred. The dispersion of drug and lipid is added to liquid nitrogen or dry ice to drop down the temperature of the sample. Afterward sample is allowed to cool at room temperature or lower temperature. The resultant powder product is SLNs (Table 7).^53^

**Table 7:** Examples of Solid lipid nanoparticles prepared by High Pressure Homogenization techniques

**HPH method**	**Drug**	**Excipient**	**Outcome**
Hot HPH	Eucalyptus globules oil	Solid lipid: cocoa butter Liquid lipids: sesame oil, olive oil, Surfactant: L-α phosphatidyl choline	Wound healing^53^
Cold HPH	Rifampicin, isoniazid, pyrazinamide	Poloxamer 188 (pluronic F-68), sodium taurocholate, stearic acid, mannitol, GMS, poloxamer 407, HPMC	As antitubercular action^54^
Hot HPH	Zataria multiflora oil	Stearic acid, tween 80, span 60, absolute ethanol	Repellant activity against anopheles stephensi^55^
Cold HPH	Streptomycin sulphate	Soy lecithin, precirol ATO 888, GMS, tween 80, PEG 400, PEG 600, Gelucire 44/14	Against mycobacterium for tuberculosis^56^
Hot HPH	Curcumin	Curcumin, Compritol 888 ATO, Soy lecithin (Phospholipon 90 G),	As wound healing^57^

###  B. Solvent evaporation/emulsification method

 In solvent evaporation/emulsification method lipophilic material is dissolved in an organic solvent and further emulsified in an aqueous phase. It forms a to give an oil in water type of emulsion.^58^ The prepared emulsion is stirred on mechanical stirrer to allow organic solvent to evaporate. SLNs are formed due to precipitation of lipid phase in water or aqueous phase. In this method polarity of two phases should be of opposite to form o/w emulsion (Table 8).^59^

**Table 8:** Examples of solid lipid nanoparticles containing drug prepared by solvent evaporation method

**Drug /compound **	**Excipients **	**Outcome **
Curcumin	Poloxamer 188, tween 80, Glyceryl monostearate, PEG-400, Ethyl alcohol	For treatment of COPD^60^
Naloxone	Glyceryl monostearate, Pluronic 127, tween 80	To inverse opioid overdose^61^
Perphenazine	Tween 80, Soy lecithin, HPLC grade acetonitrile, methanol, glyceryl monostearate	As an antipsychotic^62^
Amphotericin- B	Pluronic F 127, Vitamin B 12, Fluorescein isothiocyanate, Stearic acid, Oxyma sodium bicarbonate, Potassium bromide, phosphotungstic acid, Solutol HS 15, cellulose, Precirol ATO5	As am antileshmanial^63^
Glibenclamide	Precitrol and Compritol, PEG	For hypoglycemic effect^64^
Olmesartan medoxomil	Glyceryl Monostearate, Soya Phosphatidylcholine and Tween 80	As antihypertensive^65^

####  Limitations

1. Large amount of emulsifiers are needed to get small size particles.
2. Time and energy consuming method.
3. Solvents used if not biocompatible needs further purification is needed.

###  C. Solvent emulsification diffusion Technique

**Figure 6:**
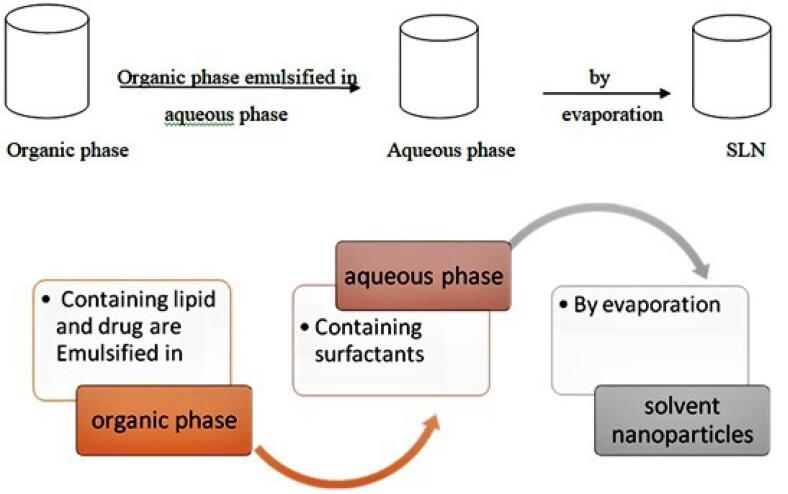
Process of solvent evaporation or emulsification method

 It consists of preparation of suspension from emulsion by a solvent diffusion technique. This process is also based on water miscibility of solvents (Figure 6). The water miscible solvents such as butyl lactate, benzyl alcohol, methyl acetate, ethyl acetate, isopropyl acetate etc are widely used. Suspensions are prepared from emulsions (with partially water miscible solvents). Process depends upon water miscibility of solvents (Tables 9 and 10).

**Table 9:** Examples of solvent emulsification diffusion technique for SLN preparation

** Drug name **	**Excipients **	**Outcome**
Tretinoin gel	GMS, Compritol 888 ATO, Dnyasan116, Cutina CBS, Epikuron 200, Tween 20, Tween 80	To treat acne^68^
Povidone – iodine gel	GMS, soyalecithin, Pluronic F 68, carbopol 940, propylene glycol	As an antiseptic drug for wound healing^69^
Rutin	Phospholipon 80 H, Tween 80, Trehalose, Ethanol, Acetate (2:1)	Use for oxidative stress induced diseases^70^
Folate conjugated Olaparib nanoparticle	PEG 4000, stannous octoate, dicyclohexylcarbodiimide (DCC), N- hydroxysuccinimide	Use as anticancer^71^

**Table 10:** Indian Patents published for solid lipid nanoparticles by various methods

**Application number**	**Year**	**Drug name**	**Title**	**Method**	**Outcome**	**Ingredients**
202111058402	2021	Lemongrass essential oil	Formulation of lemongrass essential oil loaded solid lipid nanoparticles	Hot water technique	For acne vulgaris	Oil, tween 80, ethanol, distilled water^72^
202142054086	2021	methotrexate	Surface modified methotrexate loaded solid lipid nanoparticles to overcome ABCB1 polymorphism	Microemulsion method	anticancer	Stearic acid, soya-lecithin, polyoxyethylene-polyoxypropylene^73^
202111052175	2021	Baicalein	Baicalein loaded solid- lipid nanoparticles and method of preparation of thereof	Solvent diffusion method	Neurodenegrative disorder	Baicalein stearic acid (lipid), tween80, ethanol, chloroform^74^
202141046636	2021	Clobetasol	Clobetasol loaded solid lipid nanoparticles and nanostructured lipid carriers for topical treatment of psoriasis	Melt dispersion	For psoriasis	Clobetasol, compritol, oleic acid, tween 80^75^
202121046360	2021	Sertraline hydrochloride	SLN of sertraline hydrochloride	Hot homogenization	Antidepressant and anorectic agent	Glyceryl monostearate, stearic acid, cetyl palmitate, poloxamer188, triethanolamine, ethanol^76^
202121039670	2021	Aceclofenac	Development of nanoparticle formulation of aceclofenac	Solvent evaporation method		Aceclofenac, ethyl cellulose, chitosan, HPMC K100, poly vinyl alcohol, dichloromethane, distilled water^77^
202111036625	2021	*Citrus limetta* peel	A novel composition and process for fabrication of SLNs	Ionotropic gelling agent	Diabetic neuropathy	*Citrus limetta* peel as active pharmaceutical ingredient^78^

**Figure 7:**
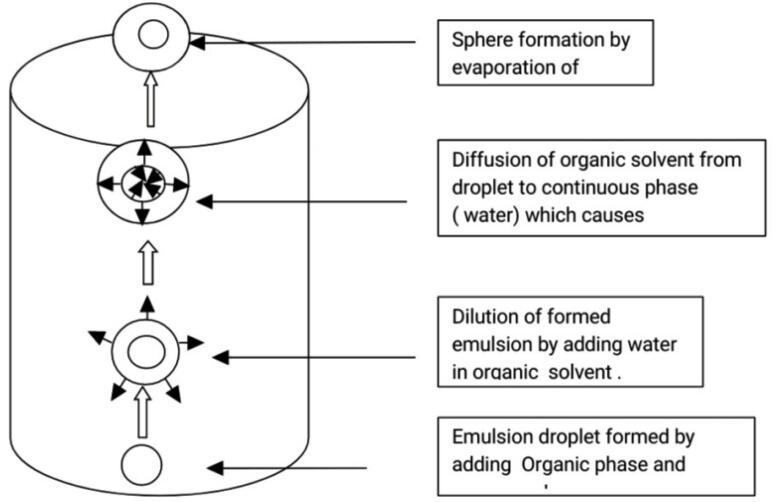
Mechanism of Sphere particle formation by solvent emulsification diffusion technique

**Figure 8:**
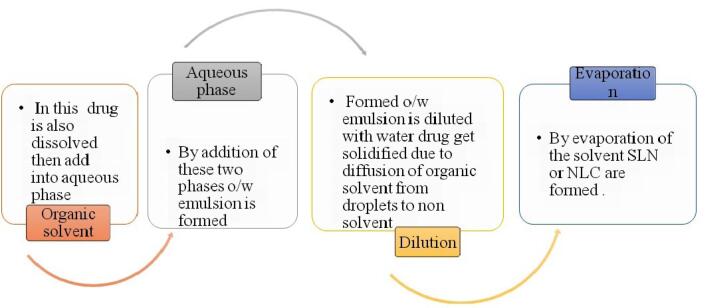
Process of solvent emulsification diffusion method for SLN or NLC

 Mechanism: It involves addition of organic phase into aqueous phase that leads to formation of o/w emulsion. Emulsion is diluted with water. During agitation provided by mechanical stirrer, dissolved drug in organic solvent gets solidified instantly due to diffusion of the organic solvent from droplets to continuous phase which forms hollow spheres (Figures 7 and 8).

#### Advantages^66^

1. The technique is easy to scale-up.
2. Exposure of drug to high temperature and physical stress will be avoided.
3. The technique is suitable for both hydrophilic and hydrophobic drugs.

#### Disadvantages^67^

1. The method requires dilution of dispersions
2. Technique requires purification process to remove residual organic solvent.

## Nanostructured lipid carriers (NLC)

**Figure 9:**
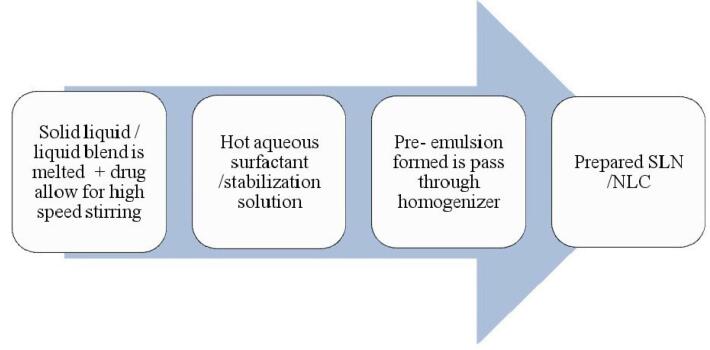
Process of Microemulsion based method for SLN or NLC

 NLC are prepared by using blend of solid lipid with a liquid lipid which remains solid at body temperature.^79-81^ The main formulation ingredients include lipids, emulsifiers and water. The preparation methods are similar to that of the SLN. SLN and NLC are similar in characteristic and techniques of preparation (Table 11). In case of SLN preparations, solid lipids are used whereas for NLC, liquid lipids or blend of solid lipid with a liquid lipid are used (Figure 9).

**Table 11:** Literature examples for development of Nanostructured lipid carrier formulations

**Technique of preparation**	**Drug used**	**Excipient used**	**Outcome**
Hot HPH	Rifabutin	Polysorbate 80, coumarin 9, glyceryldistearate (precirol ATO 5), Epikuron 145 V	Oral antimycobacterial for Crohn’s disease^84^
Sonication	Itraconazole	Precirol ATO 5, polysorbate 80, oleic acid	For pulmonary aspergillosis^85^
HPH	Tretinoin	Isopropyl myristate, Cetyl alcohol, Tween 80, Isopropyl alcohol, methyl paraben, propyl paraben.	For acne vulgaris^86^
Hot HPH	Minoxidil	Stearic acid, GMS, tripolyglycerol monostearate, oleic acid, Isopropyl myristate, Ethyl oleate, Tween 80, span 80.	For treatment of Alopecia^87^

 NLC are of three different types according to their form.^82,83^

- Imperfect type
- Amorphous type
- Multiple type

###  a. Imperfect type

 Imperfect type of NLC is prepared by different lipids with different structures and it misleads the crystal structure. This misleading can be improved with by changing saturation and number of carbon atoms in lipid. This leads to an increase in the loading capacity for drug.

###  b. Amorphous type

 Amorphous matrix is formed by mixing solid lipids with each other which forms amorphous structure.

###  c. Multiple type

 These are prepared by lipid–solid and solid-water interaction. Multiple type NLC have the advantage of increased drug loading and prolonged release of drugs due to the presence of oil droplets dispersed in solid matrix.

###  Advantages

1. Increased drug loading capacity as that of SLN.
2. Due to use of liquid mixture, differently structured molecules are formed which makes perfect crystal.
3. Perfectness of NLC system is its imperfectness for crystalline structure because they carry lattice space in between particles

## Microemulsion method

**Figure 10:**
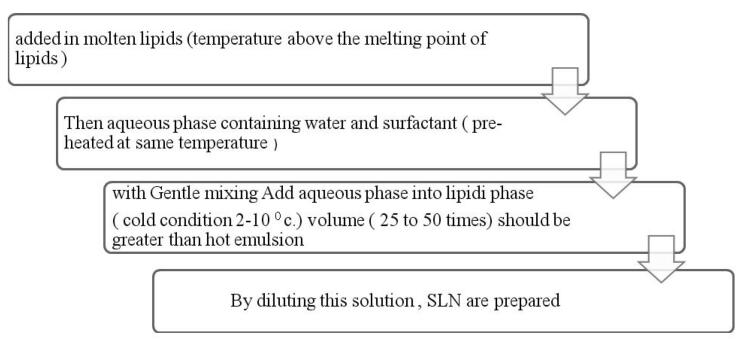
Process of Microemulsion formulation as SLN or NLCs

 These are the transparent system containing two immiscible fluids stabilized by interfacial surfactant or combinations surfactant with cosurfactants film.^88^ Microemulsions possess ultralow interfacial tension between the immiscible phases which gives thermodynamic solubility, spontaneous formation, simplicity of preparation, solubilize all lipophilic, hydrophilic and amphiphilic solutes, improve solubilisation and bioavailability of hydrophobic drugs and increases permeation (Table 12). Microemulsion method is the oil based two phasic system which contains aqueous phase and oil phase (Figure 10). Diluting microemulsion in a cold aqueous solution result in nanoemulsion then SLN/NLC prepared by lipid precipitation.

**Table 12:** Literature examples of lipid based microemulsion formulations

**Drug /compound**	**Excipients**	**Outcome**
Curcumin	Trilaurine, tristearin, triacetin, myristic acid, GMS, ethyl acetate, benzyl alcohol, polysorbate (20, 40, 80), Pluronic F68, trimyristin	Use as anticancer^88^
Anthocyanine	Palmitic acid, ethanol, isobutanol, Pluronic F127, egg lecithin, Span 85	Encapsulation to preserve anthocyanins from degradation^89^
Artesunate	GMS, Tween 80, n-butanol, ethanol	To measure intestinal permeability^90^
Rifampicin	Stearic acid, Poloxamer 407, Lipoid s-100	Use for infection of brucellosis^91^
Silymarin	GMS, Tween 80, stearic acid, cetostearyl alcohol, chloroform, propyl paraben, methyl paraben, methanol, triethanolamine, glycerol	For photoprotective activity^92^

###  Advantages

1. Thermodynamically stable, clear or colorless.
2. large scale manufacturing is possible.

Disadvantages includes requirement of high surfactant concentration.

## Hydrogel

**Figure 11:**
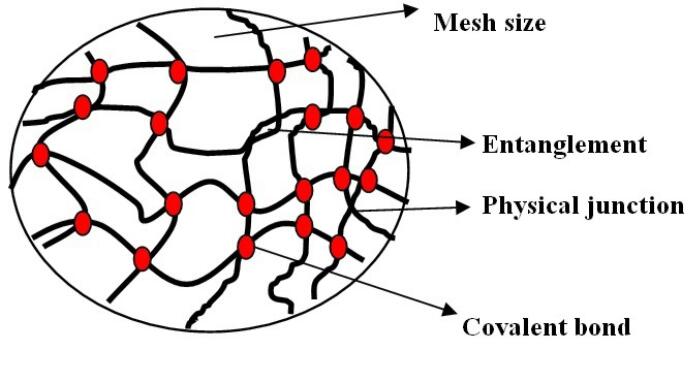
Structure of hydrogel at molecular level

 Hydrogels are three dimensional structures (formed by chemical or physical cross linking), hydrophilic and polymeric networks (cross linked monomers or chains of co-polymers) with water or biological fluid (Figure 11, Table 13).^93^ The hydrophilicity of hydrogel is due to the chemical structure of polymer backbone or group such as –OH,-COOH,-CONH,-CONH_2_,-SO_3_H and its less solubility is due to covalent bond between polymer chains or hydrophobic force, micellar packing, ionic bonding, crystallizing groups and due to presence of various bonds in the network gels.^94,95^

**Table 13:** Hydrogel types and mechanism of drug release

**Type of hydrogel**	**Mechanism of drug release**	**Polymers used**
Temperature sensitive Negatively thermosensitive Positively thermosensitive Thermoreversible gels.	Changes in temperature at various site ↓ Changes in the structure of polymer ↓ changes in swelling ↓ drug release	Poly (N-isopropylacrylamide, Poly (N,N dimethylacrylamide), Poly (N-isopropylacrylamide butyl methacrylate)^96^
pH-sensitive (acidic or basic hydrogels)	Change in PH of environment ↓ swelling ↓ drug release	Polyacrylic acid, polymethacrylic acid, polyethylacrylic acid, polypropylacrylic acid, polyhydroxyethyl methacrylate^97^
Glucose sensitive hydrogels	Glucose concentration increases ↓ swelling of hydrogel ↓ liberate drug	Hydroxyethyl methacrylate – N,N-dimethylaminoethyl methacrylate, methacrylic acid with polyethylene glycol^97^
Electric signal sensitive hydrogels	External electric field ↓ membrane charging (electrophoresis of charged drug) ↓ Swelling and drug release	Polyelectrolyte such as Polyacrylic acid co-1-vinyl 3butyl imidazole bromide (AAV), Tetraethoxysilane–fluorinated silica nanoparticles^98,99^
Light sensitive	UV radiation / visible light ↓ at fixed temperature swelling of hydrogels ↓ drug release	a. UV sensitive-triethylene tetramine b. Visible light sensitive –trisodium salt of copper chlorophyllin to poly (N-isopropyl acrylamide^100^

 Hydrogels are classified as (a) Physical hydrogels: By the formation of bonds like ionic, hydrogen or hydrophobic bonds. (b) Chemical hydrogels: Crosslinked networks, cross linking of water soluble polymers. (c) Ionic hydrogels: Polyelectrolyte are combined with multivalent ion of the opposite charge.

 Dried hydrogel also called as xero gels are more absorptive than that and called super absorbent.

## Nanocochleates

**Figure 12:**
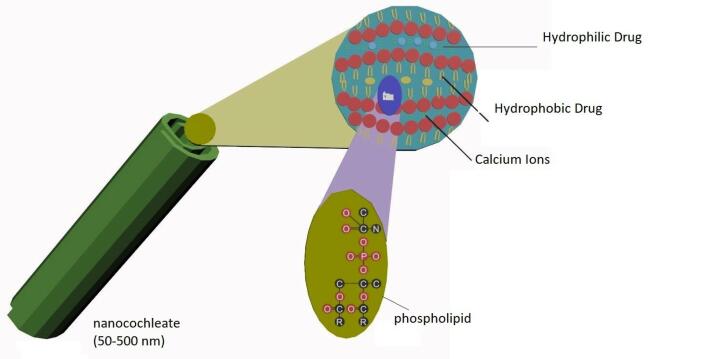
Structure of nanocochleates

 Nanocochleates are cream role like structure which is formed by the lipid bi-layers by interaction of liposomes and cations (Table 14). The sheet of phospholipids carries high tension at their edges which causes nanocochleates binding with tissue membrane (Figure 12).^101,102^

**Table 14:** Literature examples of Phospholipids and cations used for nanocochleates formulation

**Ingredients**	**Example**
Phospholipids	Phosphotidyl serine (PS) Dioleoyl phosphotidyl serine (DOPS), phosphatidic acid (PA), phosphatidyl ionositol (PI), Phosphatidoyl ethanolamine, Phosphatodoyl Glycerol (PG), Phosphatodyl choline, diolylphosphatidic acid, distearoyl phosphatidylserine, dipalmitoyl phosphatidyl glyceroyl
Cations	Zn^+2^, Mg^+2^, Ca^+2^, Ba^+2^

## Conclusion

 Novel drug delivery systems had emerged as a promising nanoplatform for efficient drug delivery. Lipid based nanoformulations was found to be beneficial to improve low aqueous solubility/poor solubility of poorly soluble drugs. The lipid based formulations have the advantage of enhancement in bioavailability for the drugs which have extensive drug The various techniques reported till today for formulation and evaluation of dosage forms as liposomes, niosomes, SLNs, nanostructured lipid carriers, nanocholates etc. Novel formulations have advantages in both solubility and permeability enhancement of poorly soluble drugs.

## Competing Interests

 All authors declare that they have no competing interests.

## Ethical Approval

 Not applicable.

## Funding

 None.
